# Do Patients’ Symptoms and Interpersonal Problems Improve in Psychotherapeutic Hospital Treatment in Germany? - A Systematic Review and Meta-Analysis

**DOI:** 10.1371/journal.pone.0105329

**Published:** 2014-08-20

**Authors:** Sarah Liebherz, Sven Rabung

**Affiliations:** 1 Department of Medical Psychology, University Medical Center Hamburg-Eppendorf, Hamburg, Germany; 2 Department of Psychology, Alpen-Adria-Universität Klagenfurt, Klagenfurt, Austria; University of Vienna, Austria

## Abstract

**Background:**

In Germany, inpatient psychotherapy plays a unique role in the treatment of patients with common mental disorders of higher severity. In addition to psychiatric inpatient services, psychotherapeutic hospital treatment and psychosomatic rehabilitation are offered as independent inpatient treatment options. This meta-analysis aims to provide systematic evidence for psychotherapeutic hospital treatment in Germany regarding its effects on symptomatic and interpersonal impairment.

**Methodology:**

Relevant papers were identified by electronic database search and hand search. Randomized controlled trials as well as naturalistic prospective studies (including post-therapy and follow-up assessments) evaluating psychotherapeutic hospital treatment of mentally ill adults in Germany were included. Outcomes were required to be quantified by either the Symptom-Checklist (SCL-90-R or short versions) or the Inventory of Interpersonal Problems (IIP-64 or short versions). Effect sizes (Hedges’ *g*) were combined using random effect models.

**Principal Findings:**

Sixty-seven papers representing 59 studies fulfilled inclusion criteria. Meta-analysis yielded a medium within-group effect size for symptom change at discharge (*g* = 0.72; 95% CI 0.68–0.76), with a small reduction to follow-up (*g* = 0.61; 95% CI 0.55–0.68). Regarding interpersonal problems, a small effect size was found at discharge (*g* = 0.35; 95% CI 0.29–0.41), which increased to follow-up (*g* = 0.48; 95% CI 0.36–0.60). While higher impairment at intake was associated with a larger effect size in both measures, longer treatment duration was related to lower effect sizes in SCL GSI and to larger effect sizes in IIP Total.

**Conclusions:**

Psychotherapeutic hospital treatment may be considered an effective treatment. In accordance with Howard’s phase model of psychotherapy outcome, the present study demonstrated that symptom distress changes more quickly and strongly than interpersonal problems. Preliminary analyses show impairment at intake and treatment duration to be the strongest outcome predictors. Further analyses regarding this relationship are required.

## Introduction

Inpatient psychotherapy, an intensive and multimodal treatment for patients with mental disorders, is especially common in Germany. There are more psychotherapeutic hospital beds and more facilities specialized solely on psychiatric disorders per capita than in any other country in the world [1]. This has also been noted in previous meta-analyses addressing special issues in inpatient psychotherapy: A large part of the included studies were conducted in Germany [2], [3]. In inpatient psychotherapy, patients are primarily treated with individual and group psychotherapy, which is complemented by other therapeutic approaches such as psychoeducational groups, occupational therapy, creative therapy, relaxation training, exercise therapy and medical treatment. Almost all psychotherapeutic hospitals offer similar complementary treatments (such as creative therapy and exercise) but the type and amount of psychotherapeutic interventions applied may vary significantly: Some hospitals offer psychodynamic, others cognitive behavioral treatment or specialized concepts (e.g. interpersonal psychotherapy or dialectic-behavioral therapy). Some clinics focus on individual sessions complemented by group sessions, while others focus on group therapy. The amount of psychotherapy ranges mainly from one to four sessions a week [1], [4]–[6].

In addition to the internationally more common inpatient psychiatric services [1], [7], there are two other treatment modalities in Germany that provide inpatient psychotherapy, i.e. psychotherapeutic hospital treatment and psychosomatic rehabilitation [5], [7]. Both treatment forms focus on psychotherapeutic rather than on medical or pharmacological approaches. In general, inpatient psychotherapy is prescribed when outpatient treatment is considered to be insufficient [5]. Accordingly, inpatient psychotherapy addresses patients at risk of self-harm, patients with difficulties to cope with everyday life or patients with serious conflicts in their social environment [5]. Hospital treatment addresses more acutely ill patients, while rehabilitation puts a special emphasis on improving patients’ working ability. Both treatment forms pursue the goals of healing patients’ disorders, preventing aggravation, or easing discomfort [8]. Schulz and Koch [8] summarize that there is a stronger indication for hospital treatment as opposed to rehabilitation in the case of curative goals, life threatening risk, profound disruptions in everyday life, a need for diagnostic assessment and a high complexity concerning medical and nursing needs. However, considering the general complexity of mental disorders, a differential allocation of patients to the appropriate acute vs. rehabilitative setting is often complicated.

In 2007, Steffanowski et al. carried out the first meta-analysis on the effectiveness of inpatient rehabilitation. This meta-analysis revealed a medium effect at discharge (overall outcome: *d*_pre-post_ = 0.57) and slightly decreased long-term effects (*d*_pre-follow-up_ = 0.49; MESTA study [9]). Given that in Germany alone, more than one million patients are treated in psychotherapeutic hospitals per year (e.g., 1,127,971 cases in 2008, [7]), it is surprising that a meta-analysis on the effectiveness of these treatments was conducted only recently by Liebherz and Rabung [10]. This meta-analysis revealed medium to large short-term effects for psychotherapeutic hospital treatment (overall outcome: *d*_pre-post_ = 0.71) with a slight increase to follow-up (*d*_pre-follow-up_ = 0.80). Due to the heterogeneity of patients, interventions, outcome measures, and study quality, the aggregated effect sizes showed a great variance [10].

In assessing the effectiveness of psychotherapy, it is essential to consider different outcome areas and outcome measures, as both may affect the results. One possibility is to distinguish between monetary (e.g. sick leave, health care utilization) and non-monetary criteria (e.g. patient satisfaction, subjective well-being) [11]. Steffanowski et al. [9] classified outcome instruments into five domains, namely physical and mental complaints, social and functional adjustment and cost effectiveness. Liebherz and Rabung [10] complemented these five domains by two other outcome areas, i.e. dysfunctional cognitive patterns and general well-being. The domain general well-being showed the highest effect sizes in the meta-analysis of Liebherz and Rabung [10] while social functioning showed the lowest. Cost-effectiveness was rarely addressed; only two studies reported results in this domain.

However, the approach ensuring the highest comparability between studies is to confine comparisons to single outcome measures. In addition, the latter solution bears the possibility of providing benchmarks to hospitals that routinely evaluate their outcomes by the use of these specific measures.

Two of the most commonly used outcome instruments in psychotherapy research are the Symptom-Checklist (SCL [12]) and the Inventory of Interpersonal Problems (IIP [13]). While the Symptom-Checklist focuses on various psychiatric symptoms (e.g. somatization, depression, anxiety), the Inventory of Interpersonal Problems deals with typical interaction problems occurring in different types of social relationships, such as being domineering/controlling or overly accommodating. Both instruments are self-rated measures.

In this context, the present meta-analysis aims to integrate all available evidence from original studies investigating the treatment effects of psychotherapeutic hospital treatment in Germany. Since it was our aim to offer specific results and benchmarks for psychotherapeutic hospitals, this paper aims to complement the first publication on this study [10], which provided data for different outcome areas over a wide range of outcome measurements. Therefore, in the present paper we confine our analyses on the two most common outcome measures used in inpatient psychotherapy outcome research, i.e. the SCL and the IIP. Additionally, we provide first results of moderator analyses.

## Materials and Methods

### Literature Search and Study Selection

We conducted an electronic database search using ‘PSYNDEXplus Literature and Audiovisual Media 1977 to September 2009’ with the following search terms (in English and German): (therapy* or treatment* or intervention*) AND (inpatient* or clinic* or hospital* or unit* or ward*) AND (result* or evaluation* or change* or effect* or efficac* or follow-up* or outcome* or course*) AND psych*. Additionally, we performed a hand search in relevant German journals (Psychotherapie, Psychosomatik, medizinische Psychologie (PPmP), Zeitschrift für psychosomatische Medizin und Psychotherapie/Psychoanalyse, Nervenarzt, Psychiatrische Praxis, Psychotherapeut, Verhaltenstherapie, Gruppenpsychotherapie und Gruppendynamik, Fortschritte der Neurologie und Psychiatrie, Forum der Psychoanalyse, Psychologische Rundschau, Schmerz, Zeitschrift für Medizinische Psychologie, Zeitschrift für klinische Psychologie, Psychopathologie und Psychotherapie, Zeitschrift für Klinische Psychologie, Psychiatrie und Psychotherapie, Zeitschrift für Psychiatrie, Psychologie und Psychotherapie) and in all reference lists of the included studies. To identify unpublished papers, we searched web pages of psychotherapeutic hospitals in Germany [14], [15]. Identified full-texts were screened for eligibility by two independent raters (SL and SR). Papers investigating the same or overlapping subgroups were integrated into one study. Only disjunctive (i.e. not overlapping) samples were considered for outcome calculation.

### Inclusion Criteria

We included published as well as unpublished papers (in German and English) reporting outcomes of psychotherapeutic hospital treatment in Germany. Investigations from other countries had to be excluded due to the differences in health care systems and the unique position of inpatient psychotherapy in Germany. Inclusion criteria were based on those used in the meta-analysis on psychosomatic rehabilitation mentioned above (MESTA study [9]), but were modified according to the context of psychotherapeutic hospital treatment (see Table 1).

**Table 1:** Inclusion Criteria (PICOS [16], [47]).

**P**articipants	Adults (18–65 years) with mental disorders (according to ICD-10, Chapter V)
**I**ntervention	Psychotherapeutic hospital treatment in Germany
	Clearly defined psychotherapy (single and/or group therapy)
**C**omparisons	Pre-assessment (start of hospital treatment) versus post-assessment (discharge)
	Pre-assessment versus follow-up-assessment (variable periods)
**O**utcomes	Symptom-Checklist (SCL) and Inventory of Interpersonal Problems (IIP)
**S**tudy design	Prospective, empirical studies
	Studies from evidence classes I (randomised controlled trials), II (quasi-experimental studies) and III (observational studies)

### Data Abstraction and Data Details

Data abstraction was mainly carried out by one rater (SL) and supported by three trained student research assistants. The student assistants extracted the sample characteristics and the quality criteria, but not the outcome data. All extracted data were verified by the first rater (SL). In case of ambiguity, the results were discussed with a second rater (SR). To guarantee a high quality of data extraction, the second rater (SR) additionally carried out unsystematic double ratings and checked for inter-rater agreement. In case of variations, which occurred in less than 1% of all ratings (for the extracted quantitative outcome data, r≥0.99), the two raters reached a consensus through discussion.

We extracted the following information from the identified studies: authors, title, year of publication, type of publication, country of study execution, study quality, measurement points, treatment characteristics (e.g. treatment duration), socio-demographic data (e.g. age, sex, education), socio-medical data (e.g. inability to work), clinical data (e.g. illness duration, diagnoses), sample size and outcome data (means and standard deviations). If relevant outcome information was missing, we contacted the authors of the study.

### Risk of Bias in Individual Studies

There is a considerable lack of available checklists for the appropriate assessment of the study quality of psychotherapy outcome studies. Especially for non-randomized, i.e. observational studies, common scales (e.g. Cochrane Collaboration’s risk-of-bias tool [16]) seem to be of limited applicability. For this reason, we scrutinized the issue of study quality in a separate study [17]. Based on a systematic review of relevant quality assessment tools, we selected the 19 different quality criteria most relevant to non-randomized psychotherapy outcome studies, which address various aspects of general methodological quality, internal validity, and external validity. To assure objectivity, we operationalized ratings as being “fulfilled” (2 points), “partially fulfilled” (1 point) or “not fulfilled” (0 points) for all items. We evaluated the quality of the included studies separately for each of these 19 criteria. Additionally, we calculated a composite score as the mean across all items, which ranged from zero to two, indicating low quality to high quality, respectively.

### Data Analysis and Data Synthesis

We calculated standardized pre-post effect sizes as well as pre-follow-up effect sizes. In the case that more than one follow-up measurement point was reported, we selected the first one following the end of treatment to calculate the pre-follow-up effect size. We conducted pre-post analyses for all subscales (a total of nine SCL- and eight IIP-subscales) and for the total scores of both instruments (SCL: Global Severity Index (GSI); IIP: Total Score). However, due to the small number of studies providing follow-up data for all subscales, we had to limit the pre-follow-up analyses to the total scores only.

Hedges’ *g* [18] was applied to correct for bias due to small sample sizes, as Cohens’ d is known to be upwardly biased when based on small samples [19] (p 48) (see Formula 1).

### Formula 1: Calculation of within-group effect sizes (Hedges’ g)



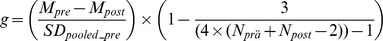



To ensure comparability across different samples (e.g. homogenous samples of depressed patients vs. heterogeneous samples with mixed diagnoses) we used the mean standard deviation pooled across all samples to calculate the treatment effect for each single sample.

According to Cohen [20], an effect of *d*>0.20/0.50/0.80 can be considered as a small/medium/large effect. However, Cohen’s classification refers to between-group effect sizes. To interpret a pre-post effect or a pre-follow-up effect as small, medium or large, this within-group effect must exceed the effect of an (untreated) control group by the reference value defined by Cohen. The mean effect size in untreated control groups in psychotherapy studies is about *d* = 0.10 [21] (p 708), [22]. Accordingly, to consider an effect size as small, medium or large, we firstly deducted 0.10 from the achieved effect sizes before applying the thresholds proposed by Cohen [20] in this meta-analysis.

To address the concept of clinical significance, we calculated the percentage of remitted patients for each sample based on the GSI of the SCL (see Formula 2). To differentiate between mentally ill and healthy subjects, we used a cut-off of *c* = 0.57 as suggested by Schauenburg and Strack [23].

### Formula 2: Calculation of remission rates



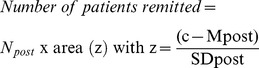
Random effects models rather than fixed effect models were applied to aggregate effect sizes across studies (see DerSimonian & Laird [24]), as they do not assume that included studies are obtained from the same population [25], [26]. By weighing each study effect with its inverse variance, smaller samples contributed less to the effect than larger samples. Results were tested for statistically significant differences to zero (two-tailed tests). To test for heterogeneity of effect sizes, we calculated *Q* statistics [18] as well as the *I^2^* index [27]. While the *Q* statistic tests for significant heterogeneity, the *I^2^* statistic quantifies the amount of variance attributed to differences between samples. *I^2^*>25 percent is considered to represent a small, *I^2^*>50 percent a medium and *I^2^*>75 percent a large heterogeneity [27].

### Moderator analyses

To address differences between the included studies, we performed moderator analyses by calculating meta-regressions via restricted maximum likelihood, weighted by the inverse variance of the particular criterion. We calculated univariate correlations between potential moderators (sample, intervention and study characteristics as reported in Table 2) and treatment effect (Hedges’ g) in SCL GSI respectively IIP Total and reported the standardized beta-weights and the p-values.

**Table 2:** Studies included in the meta-analysis.

Study	Source	Country	Treatment	Treatment Duration	N	Age M (SD)	% females	Disorder	M Intake(SCL GSI)#	M Intake(IIP Total)#	Hedges’ g(SCL GSI)#	Hedges’ g (IIP Total)#	Risk of Bias*
1.1.0	Sack et al. [48] (1997)	D	PD	/	73	28.3	76.5	mixed	/	/	/	/	medium
2.1.0	Benninghoven et al. [49] (2004)	D	mixed	/	84	34.2 (15.5)	85.0	mixed	2.10	2.41	0.81	0.46	medium
3.1.0	Kurth & Pokorny [50] (2005)	D	PD	70.0 (30.8)	142	34.0	66.0	mixed	/	1.65	/	0.31	medium
3.2.1	Höflich [51] (2005)	D	PD	31.5 (10.5)	82	33.1 (10.8)	80.7	mixed	1.32	/	0.96	/	low
3.2.2	Höflich [51] (2005)	D	PD	81.9 (27.3)	82	35.2 (10.9)	74.7	mixed	1.18	/	0.60	/	low
4.1.1	Beutel et al. [52] (2004)	D	mixed	/	404	38.5	65.0	mixed	1.31	/	0.94	/	medium
4.1.2	Beutel et al. [52] (2004)	D	mixed	/	63	33.70	67.0	mixed	1.20	1.55	0.67	0.31	medium
5.1.0	Fassbinder et al. [53] (2007)	D	CBT	92.0 (0.0)	50	30.5 (7.7)	88.0	Borderlinepersonality disorders	1.80	/	0.63	/	low
6.1.0	Deter et al. [54] (2004)	D	PD	20.0	35	44.0 (15.8)	69.0	mixed	1.14	/	0.52	/	medium
7.1.0	Dinger et al. [55] (2008)	D	PD	/	1.173	/	/	mixed	1.34	/	0.84	/	medium
8.1.1	Falge-Kern et al. [56] (2007)	D	CBT	49.2	42	35.8 (9.8)	62.0	mixed	/	1.74	/	0.62	medium
8.1.2	Falge-Kern et al. [56] (2007)	D	CBT	45.4	35	37.0 (11.5)	60.0	mixed	/	1.66	/	0.03	medium
9.1.0	Haase et al. [57] (2008)	D	PD	60.5 (24.5)	156	/	68.6	mixed	1.03	/	0.67	/	medium
10.1.1	Franz et al. [58] (2000)	D	PD	155.0 (105.0)	100	39.1 (11.5)	58.0	mixed	1.32	1.71	0.72	0.39	medium
10.1.2	Franz et al. [58] (2000)	D	PD	90.5 (48.9)	85	36.1 (13.1)	80.0	mixed	1.25	1.79	0.51	0.44	medium
10.1.3	Franz et al. [58] (2000)	D	PD	102.9 (65.8)	153	36.4 (10.1)	63.5	mixed	1.49	1.65	1.09	0.76	medium
11.1.0	Fricke et al. [59] (2006)	D	CBT	54.0	25	34.7 (10.6)	67.0	Obsessive-compulsivedisorders	1.30	/	0.86	/	medium
12.1.1	Geiser et al. [60] (2004)	D	PD	61.6 (16.8)	20	34.2 (9.5)	55.0	mixed	1.39	/	0.55	/	medium
12.1.2	Geiser et al. [60] (2004)	D	PD	71.4 (35.7)	29	31.3 (9.6)	82.4	mixed	1.39	/	0.65	/	medium
12.1.3	Geiser et al. [60] (2004)	D	mixed	56.7 (12.6)	25	38.6 (9.9)	64.0	mixed	1.73	/	1.47	/	medium
12.1.4	Geiser et al. [60] (2004)	D	mixed	61.6 (14.7)	84	32.3 (11.6)	85.7	mixed	1.43	/	0.95	/	medium
12.2.0	Liedtke & Geiser [61] (2001)	D	PD	73.5 (30.1)	45	32.0 (9.9)	73.3	mixed	/	1.77	/	0.15	medium
13.1.0	Hess [62] (1993)	D	PD	42.0	24	30.6 (9.1)	75.0	mixed	/	/	/	/	medium
14.1.1	Grabe et al. [63] (2008)	D	PD	68.5 (22.0)	217	38.5 (12.1)	41.9	mixed	0.90	/	0.58	/	medium
14.1.2	Grabe et al. [63] (2008)	D	PD	75.4 (29.0)	80	35.8 (11.4)	60.0	mixed	1.51	/	1.03	/	low
15.1.1	Hohagen et al. [39] (1998)	D	CBT	70.0 (0.0)	24	37.3 (10.8)	58.3	Obsessive-compulsive disorders	/	/	/	/	medium
15.1.2	Hohagen et al. [39] (1998)	D	CBT	70.0 (0.0)	25	33.8 (10.7)	60.0	Obsessive-compulsive disorders	/	/	/	/	medium
16.1.0	Huber et al. [64] (2009)	D	PD	/	338	39.5 (11.4)	75.0	mixed	1.40	1.75	0.78	0.39	medium
17.1.0	Junge & Ahrens [65] (1996)	D	mixed	66.8 (20.6)	164	39.7 (11.5)	68.9	mixed	1.21	/	0.48	/	low
17.2.0	Ziegenrücker et al. [66] (1996)	D	mixed	65.4 (20.2)	187	39.0 (11.1)	69.0	mixed	/	1.75	/	0.25	medium
18.1.1	Kech et al. [40] (2008)	D	IPT	35.0 (0.0)	40	39.5 (11.4)	68.0	Mood disorders	/	1.69	/	0.46	low
19.1.1	Schneider et al. [67] (1993)	D	PD	/	15	29.2	42.0	mixed	1.98	1.40	0.19	–0.17	medium
19.1.2	Schneider et al. [67] (1993)	D	PD	/	16	29.2	42.0	mixed	2.42	2.18	0.53	0.58	medium
20.1.1	Kirchmann et al. [68] (2009)	D	PD	79.3 (22.5)	37	42.9 (9.0)	58.0	mixed	1.14	1.69	0.52	0.44	medium
20.1.3	Kirchmann et al. [68] (2009)	D	PD	69.0 (22.5)	56	39.6 (11.2)	45.0	mixed	1.36	1.67	0.69	0.25	medium
20.1.4	Kirchmann et al. [68] (2009)	D	PD	/	39	28.6 (7.0)	69.0	mixed	1.42	1.88	0.65	0.61	medium
20.1.5	Kirchmann et al. [68] (2009)	D	PD	85.9 (22.5)	31	39.4 (10.5)	59.0	mixed	1.01	1.54	0.56	0.08	medium
20.2.0	Lobo-Drost [69] (2003)	D	PD	/	118	33.0	72.0	mixed	1.40	2.02	0.73	0.31	medium
21.1.0	Konzag et al. [70] (2004)	D	PD	72.0	225	32.0 (9.6)	71.0	Personality disorders	1.44	/	0.73	/	medium
21.2.0	Konzag et al. [71] (2006)	D	PD	84.0 (0.0)	43	/	100.0	Eating disorders	1.21	/	0.57	/	medium
22.1.1	Rabung et al. [72] (2005)	D	PD	95.8 (36.1)	679	35.2 (11.1)	67.5	mixed	1.48	1.75	0.73	0.47	low
22.1.2	Rabung et al. [72] (2005)	D	PD	55.5 (35.1)	240	35.2 (11.1)	67.5	mixed	1.50	1.74	0.76	0.31	low
23.1.1	Liebherz et al. [73] (2010)	D	PD	62.5 (15.5)	602	41.2 (14.0)	73.9	mixed	1.48	1.78	0.91	0.46	low
24.1.0	Sack et al. [74] (2003)	D	PD	72.7 (19.5)	61	28.5 (7.9)	72.1	mixed	1.41	/	0.97	/	medium
25.1.1	Liedtke et al. [75] (1993)	D	PD	56.0 (0.0)	50	26.8	77.3	mixed	/	1.53	/	–0.15	medium
25.1.2	Liedtke et al. [75] (1993)	D	PD	56.0 (0.0)	55	26.8	77.3	mixed	/	1.86	/	0.17	medium
26.1.0	Liebler et al. [76] (2004)	D	PD	93.8 (39.6)	87	38.2 (11.8)	55.2	mixed	1.69	/	0.97	/	medium
27.1.0	Schreiber-Willnow [77] (2000)	D	PD	93.8 (24.9)	60	43.5 (10.4)	58.2	mixed	1.34	1.73	0.66	0.38	low
28.1.0	Muhs [78] (1993)	D	PD	/	39	32.2 (7.3)	61.6	mixed	/	1.93	/	0.20	medium
29.1.0	Nickel & Egle [79] (2005)	D	PD	80.4 (22.5)	138	39.3 (10.8)	66.0	mixed	1.04	/	0.56	/	medium
30.1.0	Peikert et al. [80] (2004)	D	CBT	63.0 (12.3)	72	36.6 (9.4)	69.0	Panic disorder/Agoraphobia	1.37	/	0.99	/	medium
30.2.0	Gruhn [81] (2006)	D	CBT	86.6 (41.5)	145	34.3 (11.4)	63.0	Obsessive-compulsive disorders	1.19	/	0.91	/	low
30.3.1	Peikert [82] (2005)	D	CBT	57.8 (20.7)	67	37.7 (10.2)	64.7	Panic disorder/Agoraphobia	1.07	/	0.75	/	low
31.1.1	Pöhlmann et al. [83] (2009)	D	PD	65.4 (25.4)	153	35.5 (12.2)	71.3	Social phobias	1.60	/	0.91	/	medium
31.1.2	Pöhlmann et al. [83] (2009)	D	PD	65.4 (25.4)	460	35.5 (12.2)	71.3	mixed	1.15	/	0.61	/	medium
32.1.1	Leichsenring et al. [84] (2004)	D	mixed	157.4 (66.3)	75	32.4 (8.2)	100.0	Post-traumatic stress disorder	1.82	/	0.28	/	low
33.1.0	Schauenburg et al. [85] (2005)	D	PD	77.0	293	33.9 (11.9)	68.9	mixed	1.38	/	0.81	/	medium
33.2.0	Sammet et al. [86] (2004)	D	PD	(16.3)	213	33.4 (12.2)	70.9	mixed	/	1.62	/	–0.02	medium
34.1.0	Schneider et al. [87] (2006)	D	PD	69.3 (37.8)	173	37.8 (13.1)	78.0	mixed	1.32	1.55	0.63	0.27	low
35.1.0	Simson et al. [88] (2006)	D	PD	59.5	38	34.3 (13.4)	72.9	mixed	1.50	/	0.74	/	medium
36.1.1	Schellenberg et al. [89] (2004)	D	CBT	/	35	26.7 (6.3)	82.9	mixed	1.67	/	0.73	/	low
36.1.2	Schellenberg et al. [89] (2004)	D	PD	/	29	28.8 (6.6)	86.2	mixed	1.79	/	1.05	/	low
37.1.0	Spitzer et al. [90] (2008)	D	PD	44.4 (13.7)	130	39.7 (11.7)	76.9	mixed	1.67	1.60	0.84	0.16	medium
38.1.1	Zeeck et al. [41] (2009)	D	mixed	93.2 (32.7)	19	24.0 (7.6)	90.5	Bulimia nervosa	1.29	/	0.62	/	low
39.1.0	Tritt et al. [91] (2003)	D	unclear	56.6 (27.0)	5,898	41.0 (12.6)	67.0	mixed	1.24	/	0.83	/	medium
40.1.1	Tschuschke [92] (1993)	D	PD	167.8 (0.0)	7	27.1 (6.1)	57.1	mixed	1.72	/	0.71	/	medium
40.1.2	Tschuschke [92] (1993)	D	PD	183.0 (0.0)	7	29.9 (3.9)	42.9	mixed	1.90	/	0.71	/	medium
41.1.2	Uhlmann & Steinert [93] (2008)	D	CBT	43.0 (38.6)	13	31.5 (9.6)	78.1	Personality disorders	1.51	/	0.09	/	medium
42.1.0	Wöller et al. [94] (2007)	D	PD	/	89	39.6 (11.4)	33.7	mixed	1.30	/	0.75	/	medium
43.1.1	Zeeck et al. [95] (2006)	D	PD	135.1 (50.4)	11	23.2 (5.1)	92.3	Anorexia nervosa	1.21	/	0.70	/	medium
44.1.0	Schneider & Klauer [96] (2001)	D	unclear	84.0 (28.7)	199	34.2 (9.6)	64.4	mixed	1.27	1.72	0.96	0.48	medium
45.1.0	Seidler [97] (1999)	D	PD	84.0 (0.0)	52	30.6 (7.7)	72.4	mixed	/	1.91	/	0.48	low
46.1.0	Wälte et al. [98] (2000)	D	PD	66.4	152	/	/	mixed	/	1.81	/	0.45	medium
47.1.1	Zeeck et al. [99] (2004)	D	PD	79.8 (34.3)	18	27.3 (7.7)	100.0	Bulimia nervosa	1.80	/	0.88	/	medium
48.1.1	Zeeck et al. [100] (2009)	D & CH	mixed	60.9 (30.8)	251	41.0 (15.1)	75.2	mixed	1.38	1.45	0.78	0.28	medium
49.1.0	Probst et al. [101] (2009)	D	mixed	53.5 (24.8)	847	43.4 (12.4)	65.9	mixed	1.20	/	0.80	/	medium
50.1.0	Rasting [102] (2008)	D	PD	28.0 (0.0)	20	36.1	75.0	mixed	1.34	/	0.85	/	medium
51.1.0	Fenner & Strauß [103] (2004)	D	PD	92.0	37	26.0	70.3	mixed	1.42	/	0.77	/	medium
52.1.1	Aligwekwe [104] (2005)	D	PD	42.0	84	40.5 (9.7)	89.4	mixed	1.59	/	0.46	/	medium
53.1.0	Stingl et al. [105] (2008)	D	PD	/	397	35.6 (11.0)	70.3	mixed	1.27	/	0.69	/	medium
54.1.0	Kühler & Kasper [106] (2000)	D	CBT	97.6	16	32.4	81.3	Borderline personality disorders	1.71	/	0.73	/	medium
55.1.0	Krüger et al. [107] (2004)	D	PD	62.7 (23.8)	542	41.0 (13.3)	72.7	mixed	1.59	1.81	0.95	0.42	low
56.1.1	Bauer [108] (2004)	D	PD	130.2 (61.7)	157	34.6 (11.8)	66.0	mixed	1.23	/	0.46	/	medium
56.1.2	Bauer [108] (2004)	D	PD	134.9 (67.3)	106	36.8 (13.2)	66.0	mixed	1.32	/	0.43	/	medium
56.1.3	Bauer [108] (2004)	D	PD	132.2 (59.6)	109	33.8 (11.1)	73.8	mixed	1.33	/	0.49	/	medium
56.1.4	Bauer [108] (2004)	D	PD	137.6 (66.2)	121	35.2 (11.4)	78.5	mixed	1.18	/	0.48	/	medium
56.1.5	Bauer [108] (2004)	D	PD	120.6 (58.6)	104	36.1 (12.3)	74.5	mixed	1.29	/	0.51	/	medium
56.1.6	Bauer [108] (2004)	D	PD	142.9 (54.2)	107	33.0 (12.6)	70.8	mixed	1.25	/	0.37	/	medium
57.1.1	Schön-Kliniken [109] (2009)	D	mixed	/	16	/	/	Somatoform disorders	1.25	/	0.52	/	medium
57.1.2	Schön-Kliniken [109] (2009)	D	CBT	/	13	/	/	Somatoform disorders	1.09	/	0.66	/	medium
57.1.3	Schön-Kliniken [109] (2009)	D	CBT	/	86	/	999.00	Reaction to severe stress, and adjustment disorders	1.13	/	0.47	/	medium
57.1.4	Schön-Kliniken [109] (2009)	D	CBT	/	128	/	999.00	Somatoform disorders	1.07	/	0.53	/	medium
58.1.1	Schreiber-Willnow & Mattke [110] (2002)	D	PD	78.4 (10.0)	17	41.0 (9.4)	56.5	mixed	1.10	1.77	0.48	0.62	medium
58.1.2	Schreiber-Willnow & Mattke [110] (2002)	D	PD	96.2 (19.4)	17	39.5 (9.4)	63.6	mixed	1.20	1.73	0.57	0.64	medium
58.1.3	Schreiber-Willnow & Mattke [110] (2002)	D	PD	89.7 (19.1)	8	45.9 (9.4)	50.0	mixed	1.44	1.84	0.94	0.58	medium
59.1.1	Zeeck et al. [111] (2003)	D	PD	80.9 (51.1)	118	35.1 (13.4)	69.3	mixed	1.40	/	0.60	/	medium

# Publications with missing data in these columns did only report outcome on subscales of the SCL or the IIP.

*The risk of bias rating is based on the assessment of study quality across 19 relevant quality criteria (with ratings of 0 = not fulfilled to 2 = fulfilled). A low study quality (*M* = 0.00–0.49) indicates a high risk of bias, whereas a medium study quality (*M* = 0.50–1.49) indicates a medium and a high study quality (*M* = 1.50–2.00) indicates a low risk of bias.

### Risk of Bias across Studies

To reduce the risk of bias, we included published as well as unpublished studies. Due to difficulties in identifying unpublished studies, we also calculated Egger’s test [28] and provided the standardized beta-weights (*B*) For this test we considered the results with a *p*-value of *p*<0.10 (two-tailed) as significant to estimate publication bias conservatively – as recommended by Egger et al. [29]. Positive correlations between the standard error and the effect size indicate a “small study bias” while negative correlations indicate that small studies show lower outcome values.

### Software

For all calculations we used SPSS 15.0 [30], supplemented by a macro for meta-analysis by David B. Wilson [31].

## Results

### Study Selection

**Figure 1:**
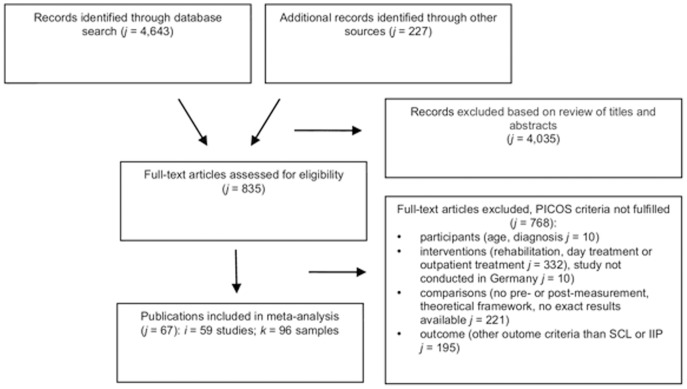
Study Flow Chart.

Based on the inclusion criteria, our search resulted in 59 studies which were described in 67 different publications (see Figure 1 and Table 2). For 34 articles of which results were incomplete, we contacted the authors. 30 authors answered, of which 20 were able to provide the relevant information. Some studies (“i”) were described in several publications (“j”, cf. Figure 1). Since some publications describe more than one sample (for example different diagnostic groups or samples which received different treatments) and do not report data for the total sample, the total number of extracted samples is *k* = 96. All samples, which received psychotherapeutic treatment and had available outcome data were included.

### Study and Publication Characteristics

Except for one study conducted in Germany and Switzerland, all studies were conducted exclusively in Germany. The majority of papers (85.1%) were published after 1999 and all others were published from 1993 to 1999. Seventy-five percent of the studies were published in scientific journals, 15 percent in books or book chapters, nine percent were not formally published and one study was published as a scientific report.

### Sample Characteristics

The majority of samples were recruited from psychodynamic treatment settings. The mean treatment duration ranged from 20 to 183 days (*M* = 80.33, *SD* = 33.07, Median = 72.70), follow-up duration ranged from three to 41 months (*M* = 13.22, *SD* = 8.09, Median = 12.00). Socio-demographic, socio-medical and clinical characteristics were typical for inpatient psychotherapeutic samples in Germany. Depressive disorders were the most common diagnoses (see Table 3). For 21.9 percent of all samples, some information about psychopharmacological treatment was available. In almost all of these samples, some – but not all – patients used medication if indicated. In one sample, patients had to be medication-free before inclusion and received a pharmacological placebo during their inpatient stay.

**Table 3:** Sample characteristics.

	SCL	IIP
Treatment characteristics	*k* = 80 samples	*k* = 37 samples
Therapeutic approach		
psychodynamic	70.0%	78.4%
(cognitive) behavioural therapy	13.8%	5.4%
mixed (e.g. psychodynamic individual sessions and cognitive behavioural group sessions)	13.8%	10.8%
interpersonal psychotherapy	0.0%	2.7%
unclear	2.5%	2.7%
**Treatment duration**		
days, *M* (SD), Median, range of means	83.5 (34.1), 78.4, 20–183^1^	74.2 (23.7), 69.7, 35–155
**Follow-up duration**	***k = *** **22 samples**	***k = *** **8 samples**
months, *M* (*SD*), Median, range of means	13.2 (7.8), 12.0, 3–41	14.1 (7.5), 12.0, 3–24
**Socio-demographic and socio-medical characteristics**	***n*** ** = 20,330 patient**	***n*** ** = 5,508 patients**
**Sex and age**		
sex (% females)	68.3%	69.8%
age (years) *M* (*SD*), range of means	38.9 (12.3), 23–46	37.4 (12.1), 27–46
**Marital status**		
single	42.5	46.8%
married	34.4	32.2%
separated/divorced	19.8	18.8%
widowed	2.7	2.6%
**Partnership**		
with partner	52.3%	50.9%
without partner	39.8%	41.5%
**Education**		
university-entrance diploma	36.4%	37.5%
**Employment**		
employed	48.6%	48.4%
unemployed	20.2%	16.6%
retired, housewife/househusband, student/trainee	23.3%	24.6%
**Clinical Characteristics**	***n*** ** = 20,330 patients**	***n*** ** = 5,508 patients**
**Illness duration (years)**		
*M* (*SD*), range of means	5.7 (7.5), 2–11	/
**Main diagnoses (ICD-10)**		
Organic, including symptomatic, mental disorders (F0)	0.1%	0.1%
Mental and behavioural disorders due to psychoactive substance use (F1)	0.4%	0.0%
Schizophrenia, schizotypal and delusional disorders (F2)	0.5%	0.1%
Mood (affective) disorders (F3)	42.7%	43.7%
Manic episode or Bipolar affective disorder (F30, F31)	0.6%	0.3%
Depressive Disorders (F32, F33, F34.1)	42.8%	43.4%
Neurotic, stress-related and somatoform disorders (F4)	33.7%	28.7%
Anxiety disorders (F40–F41)	15.4%	13.3%
Agoraphobia/Panic disorder (F40.0, F41.0)	9.6%	5.2%
Obsessive-compulsive disorder (F42)	3.6%	0.5%
Reaction to severe stress, and adjustment disorders (F43)	8.3%	3.8%
*Acute stress reaction/Post-traumatic stress disorder (F43.0, F43.1)*	3.6%	1.5%
Adjustment disorder (F43.2)	3.8%	3.0%
Dissociative (conversion) disorders (F44)	0.7%	0.6%
Somatoform disorders (F45)	7.7%	10.5%
Behavioural syndromes associated with physiological disturbances and physical factors (F5)	9.6%	9.8%
Eating disorders (F50, E66)	8.6%	11.3%
Disorders of adult personality and behaviour (F6)	12.7%	14.3%
Other mental disorders (F7, F8, F9)	0.1%	0.0%
**Comorbidity**		
Somatic comorbidity	41.9%	41.8%
Psychiatric comorbidity	69.0%	80.1%
**Impairment at intake (** ***M*** **, ** ***SD*** **, Range of means)**	***n*** ** = 17,128 patients**	***n*** ** = 5,053 patients**
Symptoms (SCL GSI)	1.31 (0.67) 0.90–2.24	/
Interpersonal Problems (IIP Total)	/	1.74 (0.52) 1.40–2.41

1 Values refer to the available data. The majority of studies provide data concerning treatment characteristics as well as basic population characteristics (sex and age). About half of the studies provide data concerning marital status, education and diagnoses. However, there is a lack of data on partner status, employment situation, illness duration and comorbidity. Only one quarter or less of all studies provides this relevant data.

### Quality Criteria

**Figure 2:**
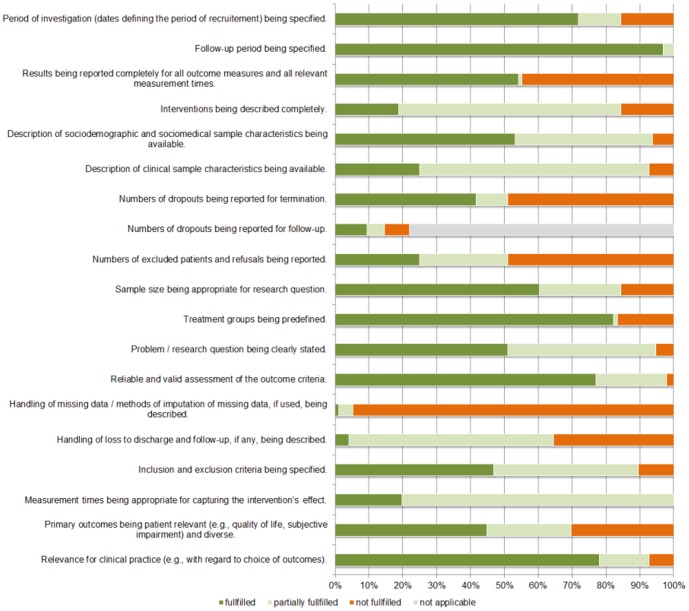
Distribution of quality criteria (k = 96 samples).

The mean quality score ranged from 0.50 to 1.78 (*M* = 1.24, *SD* = 0.27). Some criteria (i.e. definition of follow-up period) were fulfilled in almost all studies, while others (i.e. description of missing data handling) were rarely fulfilled (see Figure 2). Five percent of the studies used randomized controlled designs, 29 percent used quasi-experimental designs and 66 percent used observational designs.

### Outcome: Symptom Severity

Treatment effects on global symptom severity (GSI of the SCL) had a medium size at discharge (see Table 4) as well as at follow-up, although there was a slight reduction in effect size to follow-up (see Table 5). Taking into account the defined critical values, four percent of all samples showed no meaningful improvement at discharge, 28 percent showed an improvement of a small effect size, 49 percent showed an improvement of a medium effect size and 20 percent showed an improvement of a large effect size. No sample showed an aggravation of symptoms. Mean effects on the SCL subscales ranged from *g* = 0.46 (‘Anger/Hostility’) to *g* = 0.84 (‘Depression’). All mean effects differed significantly from zero.

**Table 4:** Pre-post-effect sizes (SCL & IIP), heterogeneity and publication bias (Egger’s test).

Scale		Outcome	Heterogeneity		Egger’s Test	
	Number of samples (*k*)	Hedges’ *g* effect size (95% CI)	*Q*	*I^2^*	*B*	*p*
**SCL**						
Somatization	36	0.48*** (0.44–0.52)	38.73	10%	–0.13	0.456
Obsessive-Compulsiveness	32	0.71*** (0.64–0.77)	72.84***	57%	–0.35	0.047
Interpersonal Sensitivity	30	0.64*** (0.59–0.69)	40.86	29%	–0.31	0.092
Depression	35	0.84*** (0.78–0.90)	76.41***	56%	–0.46	0.005
Anxiety	37	0.62*** (0.57–0.67)	54.77**	34%	–0.12	0.492
Anger/Hostility	30	0.46*** (0.40–0.51)	57.35***	49%	–0.38	0.038
Phobic Anxiety	35	0.53*** (0.45–0.60)	116.57***	71%	0.18	0.316
Paranoid Ideation	29	0.48*** (0.42–0.54)	53.01**	47%	–0.64	<0.001
Psychoticism	27	0.54*** (0.52–0.57)	22.06	0%	–0.15	0.464
Global Severity Index (GSI)	80	0.72*** (0.68–0.76)	154.09***	49%	–0.38	<0.001
**IIP**						
Domineering/Controlling (PA)	25	0.06* (0.01–0.11)	28.55	6%	–0.12	0.567
Vindictive/Self-centered (BC)	25	0.10* (0.02–0.18)	61.88***	61%	–0.15	0.485
Cold/Distant (DE)	25	0.17*** (0.11–0.22)	34.11	30%	–0.20	0.349
Socially Inhibited (FG)	25	0.36*** (0.31–0.41)	31.33	23%	–0.37	0.069
Non-assertive (HI)	25	0.32*** (0.26–0.38)	37.08*	35%	–0.32	0.114
Overly Accommodating (JK)	25	0.32*** (0.25–0.39)	44.23**	46%	–0.22	0.297
Self Sacrificing (LM)	25	0.28*** (0.21–0.35)	46.96**	49%	–0.25	0.237
Intrusive/Needy (NO)	25	0.20*** (0.14–0.27)	39.62	39%	–0.02	0.911
Total	37	0.35*** (0.29–0.41)	61.46**	41%	–0.19	0.252

*/**/*** = *p*≤0.05/0.01/0.001.

**Table 5:** Pre-post-follow-up-effect sizes (SCL & IIP).

Scale	Number of samples (*k*)	Hedges’ *g* effect size pre-post (95% CI)	*Q*	*I^2^*	Hedges’ *g* effect size pre-follow-up (95% CI)	*Q*	*I^2^*
SCL GSI	21	0.76*** (0.68–0.84)	34.51*	42%	0.61*** (0.55–0.68)	24.73	19%
IIP Total	8	0.35*** (0.25–0.45)	12.04	42%	0.48*** (0.36–0.60)	17.13	59%

*/**/*** = *p*≤0.05/0.01/0.001.

### Outcome: Interpersonal Problems

Regarding interpersonal problems (Total Score of the IIP), an improvement of a small effect size was found at discharge (see Table 4), which slightly increased but remained a small effect size at follow-up (see Table 5). Follow-up measurement points for these samples ranged from three to 24 months (*M* = 14.14, *SD* = 7.49, Median = 12.00). While 35 percent of all samples showed no substantial change at discharge (*g*<0.30), 65 percent showed improvement with 51 percent of these achieving a small and 14 percent a medium effect size. Improvement on all subscales differed significantly from zero and ranged from *g* = 0.06 (‘Domineering/Controlling’) to *g* = 0.36 (‘Socially Inhibited’).

### Outcome: Remission rates

Considering the cut-off score for the GSI of the SCL as provided by Schauenburg and Strack [23], 36 percent of patients (range: 0–56%) had achieved remission at discharge. Referring to samples with follow-up data, 36 percent (range 0–51%) had achieved remission at discharge and 32 percent (range 0–41%) had achieved remission at follow-up.

### Heterogeneity

Concerning pre-post effects, 12 of 19 scales (i.e. 63%) showed significant heterogeneity (Q-score p<0.05, see Table 4). Heterogeneity (I^2^) ranged from 0% (SCL ‘Psychoticism’) to 71% (SCL ‘Phobic Anxiety’). Eleven scales (58%) showed small (*I^2^* = 25–49%) and four scales (21%) showed medium heterogeneity (*I^2^* = 50–74%). No scale revealed large heterogeneity (*I^2^*>75%).

Pre to follow-up effects showed no significant heterogeneity, however the I^2^ score for the IIP Total indicated a small amount of heterogeneity. The SCL GSI’s I^2^ at follow-up was smaller than 25 percent, indicating low heterogeneity (see Table 5).

### Moderator analyses

To explain the heterogeneity in treatment effects, we examined the percentage of females, mean age, diagnostic composition (homogeneous vs. heterogeneous diagnostic groups), impairment at intake, type of treatment (cognitive-behavioural, psychodynamic or mixed), treatment duration, publication year as well as the mean study quality as potential moderators. In both SCL GSI and IIP Total, only impairment at intake (SCL GSI: *β_standardized_* = 0.28; *p* = 0.013; IIP Total *β_standardized_* = 0.35; *p* = 0.031) and treatment duration (SCL GSI: *β_standardized_* = –0.43; *p*<0.001; IIP Total *β_standardized_* = 0.41; *p* = 0.015) achieved significance. While a higher impairment at intake was associated with a larger effect size in both measurements, a longer treatment duration correlated with lower effect sizes in SCL GSI and with larger effect sizes in IIP Total.

### Risk of Publication Bias

**Figure 3:**
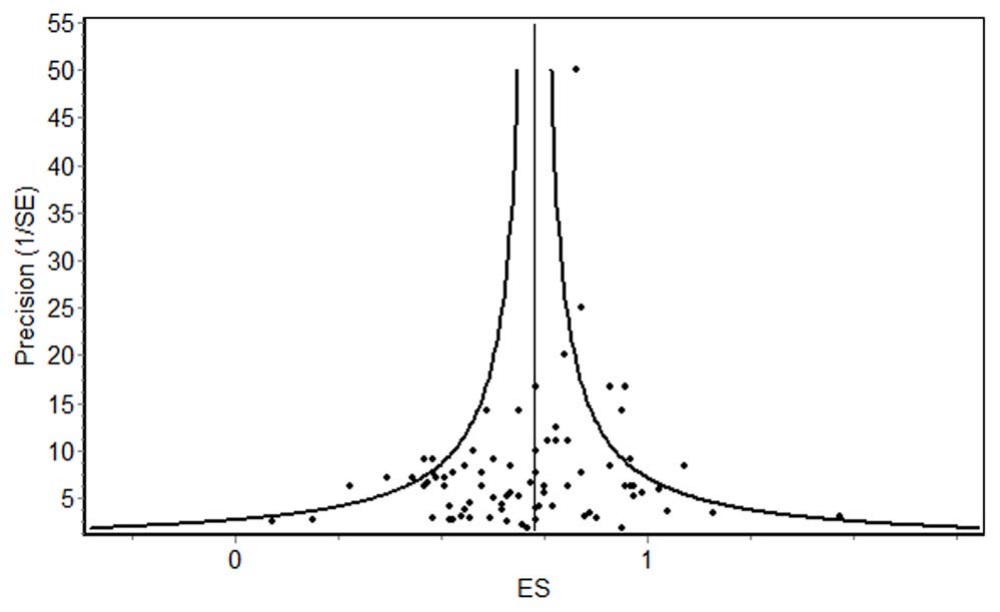
Funnel plot SCL GSI (k = 80 samples).

We calculated the risk of publication bias with the Egger’s test. Results showed a significant asymmetry (*p*<0.10) regarding the SCL’s GSI, the SCL’s subscales ‘Obsessive Compulsiveness’, ‘Interpersonal Sensitivity’, ‘Depression’, ‘Anger/Hostility’ and ‘Paranoid Ideation’ as well as for the IIP subscale ‘Socially Inhibited’ (see Table 4). In all of these scales, smaller studies showed lower effects (see Figure 3).

## Discussion

This study represents the first meta-analysis on the effectiveness of psychotherapeutic hospital treatment in Germany. There is a substantial data base of 59 included studies that applied either the Symptom Checklist (SCL [12]) or the Inventory of Interpersonal Problems (IIP [13]) as an outcome measure.

It can be concluded that psychotherapeutic hospital treatment shows positive outcomes for both psychopathological symptoms and interpersonal problems. However, the effects in the two domains differ in their magnitude and pattern. Symptom reduction reaches a medium effect size at discharge but the effect slightly decreases between discharge and follow-up. On the other hand, interpersonal problems are reduced at a slower pace and are less substantial in the short term, yet they continue to decrease from discharge to follow-up. Similar results have been reported by Barkham et al. [32] who also found higher effect sizes in symptom improvement than in interpersonal problems. These findings correspond to Howard’s phase model of psychotherapy outcome [33] that indicates three phases of outcome (i.e. remoralization, remediation and rehabilitation). According to this model, the first improvements are expected to occur in subjective well-being, which then allows for a symptom reduction. Symptom reduction on its own seems to be a necessary condition for improvement in life functioning, including interpersonal functioning.

An additional explanation for lower effect sizes concerning the IIP might be that we combined the IIP Total Score and individual subscales across all different kinds of samples. This resulted in a reduction of information, as different patient samples show different patterns of interpersonal problems. The simple mean value calculation, however, does not consider the circumplex structure of interpersonal problems [34].

Compared to the samples of inpatients treated in psychosomatic rehabilitation clinics in Germany which were included in the meta-analysis conducted by Steffanowski et al. [9], the present samples of inpatients treated in psychotherapeutic hospitals showed a higher symptom severity at intake (*M* = 1.31, *SD* = 0.67 vs. *M* = 1.14, *SD* = 0.69, d = 0.25). Correspondingly, symptom reduction at discharge (*d* = 0.76 vs. *d* = 0.66) and at follow-up (*d* = 0.62 vs. *d* = 0.46) was higher as well. However, when interpreting these differences in effect size, one needs to consider several possible reasons favoring hospital treatment. First, a higher initial symptom load may allow for a higher symptom reduction. Second, hospital treatment is characterized by a longer duration than rehabilitation treatment (twelve vs. eight weeks on average across the included studies). Third, as the present analysis was able to include more recent studies than the MESTA study, more advanced treatment concepts may in part account for the existing differences.

On the one hand, it can be concluded that there is variation regarding the effectiveness of psychotherapeutic hospital treatment differing between the included scales. The heterogeneity index varied from 0 percent (SCL ‘Psychoticism’) to 71 percent (SCL ‘Phobic Anxiety’). As we included almost no sample with psychotic patients, the high homogeneity between samples regarding ‘Psychoticism’ appears plausible. With regard to ‘Phobic Anxiety’, our results may reflect the fact that certain symptoms are more prevalent in some samples than in others. On the other hand, considering the diversity of patients and treatments in the included studies, the finding that no subscale showed a large heterogeneity (all *I^2^*<75%) indicates the presence of basic similarities in the treatments under study. Surprisingly, the follow-up results show only a small (respectively smaller) heterogeneity even though there was a high variability in follow-up intervals (ranging from three to 41 months). The variety of heterogeneity between different subscales corresponds to typical characteristics of the investigated sample.

None of the investigated patient characteristics except the impairment at intake correlated significantly with the treatment effect. In accordance with Bohart and Greaves Wade [35], samples with higher impairment at intake show larger changes during treatment. Concerning treatment characteristics, only the treatment duration was associated with the effect sizes; interestingly, the results differed between the two investigated outcome measurements. With regard to symptom severity, longer treatment duration is associated with lower effect sizes, whereas regarding interpersonal problems, samples with longer treatment durations showed larger effect sizes. Generally, the relation between outcome and treatment duration is not a simple one: While dose-response-models [36], [37] postulate that treatment duration affects outcome (higher response rates in longer treatment), the good-enough-model [38] implies that symptom change predicts treatment duration (longer treatments in severely disturbed patients). In this context, our findings may reflect the reality that symptom change constitutes a primary outcome of inpatient psychotherapy while change in interpersonal problems constitutes a more secondary goal. However, our data do not allow for any more detailed interpretations. To clarify these relations, further studies are required.

The quality of included studies was not significantly associated to the treatment effect, accordingly there is no evidence that low quality studies overestimate the treatment effects in this meta-analysis.

One major limitation of this meta-analysis may be seen in the lack of randomized controlled trials (RCTs) addressing the efficacy of psychotherapeutic hospital treatment. This lack of RCTs may be attributed to the specialties of the German health care system and its indication standards for inpatient and outpatient psychotherapy: As inpatient psychotherapy is considered to be the indicated and available treatment option for seriously disturbed patients in Germany, an allocation to a treatment condition of lower intensity (i.e. outpatient treatment or waitlist) would be considered unethical. Therefore, any study aiming at evaluating the efficacy of psychotherapeutic hospital treatment by use of an RCT design would be disapproved by the local ethics committee. Correspondingly, the only existing RCTs in this field compare different treatment conditions within inpatient psychotherapy [39], [40] or – on rare occasions – inpatient to day clinic treatment [41].

As a consequence, our analysis had to focus on observational or quasi-experimental pre-post/pre-follow-up comparisons. Thus, this meta-analysis does not allow causal interpretations. Changes cannot exclusively be attributed to the psychotherapeutic treatment but may also be caused by spontaneous remission or other confounding influences. In addition, as psychotherapy is only one part of the multimodal inpatient treatment concept, the proportion of improvement caused by psychotherapeutic interventions in a narrower sense remains unclear. Since the application of psychopharmacological treatment is rarely described, analyses on the influence of medication were not feasible in this meta-analysis. In one of the included randomized controlled trials, the combination of behavior therapy and fluvoxamine was superior to behavior therapy and placebo in patients with obsessive-compulsive disorder regarding obsessions and depressive symptoms but not superior regarding compulsions [39]. In another randomized trial, the application of interpersonal psychotherapy additional to pharmacotherapy showed a higher reduction of depressive symptoms compared to pharmacotherapy plus clinical management, but was not superior regarding social and interpersonal functioning [40]. Cuijpers et al. [2] found a small statistically significant additional effect favoring psychological treatments compared to usual care and structured pharmacological treatment in depressed inpatients. Regarding these results, one can assume that the psychotherapeutic treatment itself is an effective factor in this setting - at least in some outcome areas.

Schauenburg and Strack [23] reported data for the SCL’s GSI in large German psychotherapy samples (outpatients, *M* = 1.12, *SD* = 0.57; inpatients *M* = 1.29, *SD* = 0.70). In accordance with the indication for inpatient psychotherapy, patients in our sample show higher impairment at intake than outpatients (SCL GSI: *M_pre_* = 1.31, *SD_pre_* = 0.67; d = 0.31). At discharge, the patients we studied were less disturbed than typical outpatients (SCL GSI *M_post_* = 0.79, *SD_post_* = 0.60; d = −0.56) but still more than twice as impaired as the German norm population (*M* = 0.33, *SD* = 0.25, d = 1.08 [23]). Still, one third (36%) of the examined patients reached remission.

To date, established criteria to classify within-group (e.g. pre-post) effects are lacking. We addressed this problem by deducting the effect sizes occurring in untreated control groups in (outpatient) psychotherapy studies [21], [22] from our calculated effect sizes before applying the critical values which have been proposed by Cohen for the interpretation of between-group effects [20]. However, this provisional approach certainly requires further validation.

A possible imprecision of effect size calculation could as well have arisen from lacking information about pre-post-correlation in outcome measures, which did not allow the consideration of interdependence [25].

Methodological weakness of included studies is often criticized as one major source of bias in meta-analyses. To do justice to the complex relationship between study quality and outcome of psychotherapy, we carried out an extensive complementary project on this issue [17]. Based on a comprehensive review of the literature and an expert rating, we selected 19 relevant quality criteria to quantify the quality of the included studies. With a mean score of *M* = 1.24 (*SD* = 0.27) on a scale ranging from 0 = ‘low quality’ to 2 = ‘high quality’, the overall quality of the included studies may be considered as medium. However, study quality varies considerably over different studies and different criteria. Especially in terms of dealing with dropouts, more detailed information in original papers is required. In spite of these limitations, there is no evidence that low quality studies distort the results of this meta-analysis since no correlation was found between study quality and outcome. Although our approach allows for a sophisticated appraisal of relevant quality criteria, especially with regard to non-randomized studies, there are no benchmarks available until now, since this is the first application of our checklist.

The majority of the studies’ outcome parameters showed no significant results in Egger’s test. As the few significant results indicated overally smaller effects in smaller studies, there was no evidence for a small study bias.

Some studies provided more than one publication, which complicated the process of data abstraction and data aggregation since the different publications sometimes focused on partially overlapping subgroups. We emphasized on including all relevant information without integrating data from overlapping subgroups in our calculations.

Data regarding employment status, illness duration and comorbidity were incomplete in many cases, which limited the representativeness of the overall sample description. Heterogeneous classifications of socio-demographic variables complicated a consistent data aggregation. Fortunately, at least data on the therapeutic approach, age, sex and the main diagnoses were nearly complete.

The SCL is a well-established instrument in psychotherapy research. It is able to differentiate between subjects with and without a psychiatric disorder and is qualified for measuring change in outcome studies [42]. The SCL GSI shows a high internal consistency [42], [43], while the results on the subscales are inconsistent [42], [44]. Previous studies show that most of its subscales measure one broad dimension of general symptom distress and are not suitable to differentiate between various diagnostic groups, therefore the concept of multi-dimensionality is doubtful [42]–[46]. The IIP scales are dominated by this general factor as well, but also showed high loads on three factors on interpersonal behavior and interpersonal problems identified by Tran et al. [43]. On the one hand, it is therefore questionable whether the IIP provides relevant additional information. On the other hand, our results show different results in IIP compared to SCL, justifying the application of both measures. Even if the factorial validity of the subscales is doubtful, we reported these results to provide benchmarks for facilities applying these scales for evaluation purposes.

As the high number of included studies involved an immense effort regarding the data extraction, results of this extensive meta-analysis were not available until more than four years after the end of the literature search. Although we expect some relevant studies to be published during this period, the included studies may still be regarded as being up-to-date. Due to the large number of included studies, we do not expect that a small number of new studies would change the results significantly.

Due to the restriction of the electronic literature search to the primarily German database PSYNDEX, some exclusively English publications may have been missed. However, this risk of bias can be assumed to be low as PSYNDEX comprises more than 500 English journals and electronic search was complemented by a comprehensive hand search.

## Conclusion

In spite of all methodical limitations in this meta-analysis, there is evidence that psychotherapeutic hospital treatment shows positive outcomes regarding symptom severity as well as interpersonal problems in severely disturbed patients. To clarify the relations between symptom severity, interpersonal problems and treatment duration, further research is required.

## Supporting Information

Checklist S1 **PRISMA 2009 Checklist [112].**
(DOC)
